# Statistical Assumptions and Reproducibility in Psychology: Data Mining Based on Open Science

**DOI:** 10.3389/fpsyg.2022.905977

**Published:** 2022-05-30

**Authors:** Wenqing Zhang, Shu Yan, Bo Tian, Dingzhou Fei

**Affiliations:** Department of Psychology, Wuhan University, Wuhan, China

**Keywords:** reproducibility of psychology, data mining, normality hypothesis, homoscedasticity hypothesis, robust hypothesis

## Abstract

The failures of reproducibility in psychology (or other social sciences) can be investigated by tracing their logical chains, from statistical hypothesis to their conclusion. This research starts with the normality hypothesis, the homoscedasticity hypothesis, and the robust hypothesis and uses the R language to simulate and analyze the original data of 100 studies in *Estimating the Reproducibility of Psychological Science* to explore the influence of the premise hypothesis on statistical methods on the reproducibility of psychological research. The results indicated the following: (1) the answer to the question about psychological studies being repeatable or not relates to the fields to which the subjects belonged, (2) not all the psychological variables meet the normal distribution hypothesis, (3) the *t*-test is a more robust tool for psychological research than the Analysis of Variance (ANOVA), and (4) the robustness of ANOVA is independent of the normality and variance congruence of the analyzed data. This study made us realize that the repeatable study factors in psychology are more complex than we expected them to be.

## Introduction

Whether a particular research study can be reproduced or not, is a vital indicator to evaluate the reliability of scientific research (Schmidt, 2009). For a long time, however, there was no proper way to solve the reproducibility problem in psychology. In 2011, Simmons et al. (2011) conducted a rather comprehensive analysis of false positives in a psychological study. The findings of this analysis made a certain impact on the psychological circles. Next year, several psychologists (Simmons et al., 2011; Galak et al., 2012; Ritchie et al., 2012) repeated a study by Bem (2011). Their experiment did not obtain the same results as the original study, which set off a heated discussion and resulted in an upsurge of research on the reproducibility problem in psychology. Since then, many researchers have begun to carry out repetitive research work on some of the classic psychological experiments. In many of these cases, a repeat of the results of the original study did not occur.

Since it is difficult for a single team to carry out large-scale repetitive experiments on psychological research, the Open Science Center (OSC) has been established to facilitate such research by combining the efforts of psychologists from around the world. However, in many of the replication projects that have been carried out, the results have not been uniform. Some projects have had a high reproduction rate, while others have not (Barnett-Cowan, 2012). For example, the Many Labs Project conducted by OSC in 2014 that selected 13 classic social psychological effects for repetition found that 11 effects were repeated (Open Science Collaboration, 2015), which is a high reproduction rate. However, the success rate of the Reproducibility Project of Psychology (RPP), was only 39%. Further, the results of the 64 repeated studies were not significant and in the 36 successfully repeated studies, the average validity was somewhat lower than in the original study (Klein et al., 2014).

It is generally believed that the difficulty in reproducing the earlier results in the replications of psychological research is caused by several reasons, which are mentioned below.

### Uncertainty of the Statistical Significance Test

For psychological statistics, it is generally believed that when *p* < 0.05 (or 0.01), the result attains significance. However, there are logical flaws in applying this statistical method. These principal flaws in statistical tests may attribute to the misunderstandings of the *p*-value. On other hand, currently, statistical education for researchers and students over the world would be partly responsible too. Other logical flaws were pointed out to be violating the modus tollens, a valid logical rule (Trafimow, 2019a).

For a given hypothesis test, data is obtained from experiments, H0 and H1 are null and alternate hypotheses, respectively. When we select a value α in [0,1] randomly, the logic of NHST is essentially an argument by contradiction, designed to show that the null hypothesis will result in a non-sensical conclusion and thus must be rejected, so, a hypothesis test under a reject area W and a significant level α, aims to check if


(1) $$\begin{array}{l} \text{Pr }(\text{D}\epsilon \text{W}|\text{H}0)\leq \text{α}. \end{array}$$

While what we want to know for *p*-value is: given these data, the probability that H0 is false or falls in the reject area W is **Pr** (**rejectH0|D**). This *p*-value is set to the minimal value for comparison with the significant level α to make the final judgment. But what the hypothesis test actually says is the probability of these data occurring given that H0 is true, **Pr**(**Dϵ** **W |H0**). As Cohen pointed out in 1994, the two are different things [Cohen, 1994. The earth is round (*p* < 0.05). American Psychologist, 49, 997–1003]. The *p*-value from the statistical test does not tell us the answer we want.

The conditional probability **Pr** (observation | hypothesis) ≠ **Pr** (hypothesis | observation)

Using the *p*-value as a decision score is a logical error: the transposed conditional fallacy.

From the view of the formal logic, this logical error has another version of a logical fallacy for conditional reasoning, named “fallacy of affirming the consequent”.

The scheme (if …. then conditionals) “Because (if A then B), then can deduce (if B then A)” is not valid reasoning. we now have A = observation, and B = hypothesis, if using *p*-value as the usual way in NHST, we committed to **Pr** (**observation** | **hypothesis**) = **Pr** (**hypothesis** | **observation**), then we have made a logical mistake, i.e., the fallacy of affirming the consequent. These fallacies are violating a valid rule: modus tollens (MT).

Our view on *p*-value is consistent with the American Statistical Association's statement on the understanding of *p*-values (Wasserstein and Lazar, 2016), which includes the followings: “(1) *p*-value can indicate how incompatible the data are with a specified statistical model. (2) *p*-value neither measures the probability that the studied hypothesis is true nor the probability that the data were produced by random chance alone. (3) Scientific conclusions and business or policy decisions should not be based only on whether a *p*-value passes a specific threshold. (4) Proper inference requires full reporting and transparency. (5) A *p*-value, or statistical significance, does not measure the size of an effect or the importance of a result. (6) By itself, a *p*-value does not provide a good measure of evidence regarding a model or hypothesis.”

There are debates on *p*-value, and mainly focus on how *p*-value is retained and modified, and whether alternatives are feasible (for example, the Bayes factor) or even abolished *p*-value. It still is widely utilized in social sciences.

### Sample Limitations

To some extent, the sample problem is rooted in the statistical significance test, which is highly dependent on the sample size. However, the reality is that psychological researchers often choose a small sample size not exceeding 30 persons in consideration of the time limitations and economic costs, even though a larger sample will be more representative. Additionally, most of the present-day studies are based on college students (Cohen, 1995), when college students should constitute only a sub-group of 5%. This raises further doubts about the representativeness of the current studies and poses a risk to the validity of their results. There are disputes about avoiding a small population to affect the validity of psychological studies, and if we do not adopt *p*-value (we just state that the *p*-value is doubtful in the last passage), then we have an alternative way to estimate the population necessary to the hypothesis, for example, a priori procedure (APP) is able to help to determine the minimum sample size to reach to precision and confidence (Trafimow and Myuz, 2019).

### Effectiveness of Statistical Methods and Models

To ensure the validity of statistical testing, it is generally necessary to verify that the distribution of data is normal before statistical methods are applied. However, researchers who examined 513 papers published in the Journal of Psychology found that of these 157 papers the authors described at least one case in which statistical errors were included, and in these 157 papers, 79 papers included at least one or more statistical errors (Open Science Collaboration, 2015). This finding undoubtedly questions the credibility of the results of these studies.

### Flexible Experimental Design and Selective Report

Overemphasis on the *p*-values in the current replication studies has directly led to blind obedience to the significance test among the psychological researchers concerned. Sometimes, the researchers manipulate their experiments to a certain extent in terms of selecting the sample source or the sample size to increase the probability of significance and partially report the experiment's data. If the researchers manipulate all these factors, then the probability of a positive significance (*p* < 0.05) could be more than 61% (Simmons et al., 2011).

Non-reproducibility is also related to several issues of statistical analysis. For example, it cannot be said with certainty that while conducting psychological statistical analysis the theoretical basis of the hypothesis test and the premise of the use of the central limit theorem (i.e., the independence of observations) has been satisfied. Secondly, given the particularity of the psychological research object, can we say that its objectivity and measurement stability have been guaranteed? These issues cannot be ignored.

In our study, before the preliminary test, a rough distinction was made between the psychological variables of 100 studies following the methods mentioned in a study *Estimating the Reproducibility of Psychological Science*. According to the types mentioned in the journal, these studies were categorized in terms of their empirical basis into the fields of social psychology or cognitive psychology. Based on the results of the categorization from the paper, the following hypotheses are presented:

**H1.1** Studies in cognitive psychology are more likely to be reproduced than those in social psychology.

There are some important differences between the research objects and methods of social psychology and cognitive psychology. In comparison, the objects of social psychology are more subjective (such as emotions, attitudes, and subjective reports) than ones of cognitive psychology. These differences may affect the replication of experimental outcomes.

**H1.2** Not all psychological variables in the psychological studies meet the normality hypothesis.

For example, many reaction time data is skewed rather than normal (Trafimow et al., 2019).

Two psychologists have discussed that psychological research is generally divided into three levels (Yang and Sun, 2011): firstly, the micro level, which is mainly used to study the brain or neural mechanisms, that is, the neuropsychological level to study psychological phenomena; secondly, the meso level, which is mainly used to study the behavior and psychological processes of individuals and their psychological structure and functional characteristics during development; the laws at this level are more stable. Finally, the macro level is used to investigate the relationship between people and society, or the psychological phenomena and structures in different cultural contexts, which is the social level of psychology. Cognitive psychology belongs to the meso level and social psychology to this level. This thesis suggests that there may be some important differences between the patterns followed by cognitive and social processes, with the former being closer to natural scientific features than the latter. We then have the following hypothesis **H1.3:**

**H1.3** Data in cognitive psychology studies are more likely to conform to normality assumptions.

In total, a rough count of the statistical methods was conducted for 97 studies, originally a total of 100 replicated studies, of which two studies used the same data source and two studies did not have access to the original data. Of these 97 studies, a total of six statistical analysis methods were used, with a small sample of studies utilizing chi-square tests, correlation analysis, multi-layer linear models, and hierarchical linear models (12.4% in total), so the focus was on ANOVA (62.9%) and *t*-tests (23.7%). Of these 97 studies, a total of six statistical analysis methods were used, with a small sample of studies utilizing chi-square tests, correlation analysis, multilayer linear models, and hierarchical linear models (12.4% in total), so the focus was on ANOVA (62.9%) and *t*-tests (23.7%). Table 1 provides a summary of the methods used in these 97 studies.

**Table 1:** Statistical methods used in the selected studies.

**Method**	**Cognitive psychology**		**Social psychology**		**PCT in**

	***F***	***PCT***	***F***	***PCT***	**total**
*t*-Test	12	28.5%	9	20.0%	24.1%
ANOVA	29	69.0%	30	66.7%	67.8%
Correlation	1	2.3%	4	8.9%	5.7%
HLM	0	0	1	2.2%	1.1%
LM	0	0	1	2.2%	1.1%

Relatively speaking, among the two most frequent statistical tools, the robustness of the *t*-test is stronger than that of the analysis of variance because for ANOVA both the normal distribution and the homogeneity of variance are established. We have hypothesis **H2.1:**

**H2.1** Studies using the *t*-test are more likely to be reproducible than those using ANOVA

In addition to the requirement of normal distribution, the analysis of variance also requires the homogeneity of variance, which is relatively strong. Therefore, from a logical point of view, satisfying the premise of the test can improve the repeatability of the research. So, we have the following two assumptions **H2.2** and **H2.3:**

**H2.2** The more the data of a study meet the normal distribution hypothesis of ANOVA, the greater the possibility of reproducing the study.
**H2.3** The more the data of a study meet the homogeneity of variance hypothesis of ANOVA, the greater the possibility of reproducing the study.

It is easy to overlook but important to note that the concepts of hypotheses, assumptions, and models are used extensively in this study and the reader can distinguish between them in the following ways: the statistical assumptions commonly used in our research on psychology (e.g., the normality assumption) and the research hypothesis, mainly reference to H1.1-3, and H2.1-3 above, which is the subject of this paper, and the models, which in most cases refer to statistical tools such as linear models, ANOVA, etc. However, when we test research hypotheses, we are actually discussing the overall degree of consistency of statistical assumptions, the statistical method, and the data. Since we just questioned the rationality of *p*-value suitable for hypothesis testing, if it is used for hypothesis testing, it should be understood as a test of the incompatibility of the overall hypothesis or model, rather than the usual use of *p*-value as the only basis for judgment. This dealing with *p*-values as an index for the incompatibility in a model, but not a hypothesis can avoid the conflicts about views for the *p*-values, and then in the following chapters, we can use *p*-value to make tests of hypothesis H1 and H2 series.

## Method

### Data

Since this study examines the factors that affect the reproducibility of psychological research, it was necessary to select a considered number of psychological experiments that had been reproduced to be able to examine the factors that influence reproducibility. After multiple comparisons, our study selected 100 reproduced studies (https://osf.io/hy58n, and https://osf.io/ezcui) as raw data for this study. These 100 studies were selected from three well-known psychology journals: *Psychological Science, Personality and Social Psychology, and Experimental Psychology: Learning, Memory and Cognition*. Further details about the filtering criteria and the process of selecting the repetitive studies can be found in reference (Open Science Collaboration, 2013).

For the analysis vis-a-vis the three different hypotheses, we used R for data cleaning, data simulation, and visualization of the results.

#### Methods of Normality Tests and Simulations

The most widely used normality tests based on frequency statistics are the D'Agostino's K-squared normality test, the Jarque–Bera normality test, Anderson–Darling normality test, Lilliefors normality test, Kolmogorov–Smirnov normality test, Shapiro–Wilk normality test, and Pearson's chi-squared normality test. Other normality tests such as the Jarque–Bera normality test have less validity when it comes to tail distribution especially the Twin Peaks distribution (Yap and Sim, 2011). Many researchers believe that the overall performance of the Jarque–Bera Test is too poor to apply to their research; the Lilliefors normality test is a correction of the Kolmogorov–Smirnov normality test to make the latter better suited for large samples without changing it. The disadvantages of the Kolmogorov–Smirnov normality test and D'Agostino's K-squared normality test were tested using skewness and kurtosis. The latter works better only when there is a skewed distribution or leptokurtic distribution (Romão et al., 2010). Considering the above factors, we used the Shapiro-Wilk normality test for our study. Null hypothesis testing based on frequency statistics is currently the most widely used within various disciplines (Normality Test, 2017). China's national standard for normality test, the latest version of GB/T 4882-200, was the Shapiro–Wilk normality test (Liang, 1997). Therefore, this study uses the null hypothesis test based on frequency statistics for normality testing. We use R programming to simulate the normality. In RStudio setting, we first load a dataset from OSC webs (see section Data, part of data sources) and use the function “shapiro.test (data$CreditScore)” and visualizations to fit normality. R programming will output normality test results with *p*- values. Readers can refer to source codes in R by the Supplementary Materials at the end of this study.

#### Methods of Homogeneity of the Variance Tests and Simulations

We generally believe that the significance test is invalid when the variance is heterogeneous. The methods used in the group variance test are the F-test of equality of variances, Cochran's C test, Hartley's test, and Bartlett's test. The parsley tests for all regression analyses are the Levene's Test, Goldfeld–Quandt Test, Park Test, Glejser Test, Brown–Forsythe Test, Harrison–McCabe Test, Breusch–Pagan test, White test, and Cook–Weisberg test. Considering that our study only conducts a variance homogeneity test in ANOVA analysis and does not require a variance test in regression analysis, it was compared with several commonly used test methods in ANOVA. In the several widely used methods of variance testing, Bartlett's test, F-test of equality, and Hartley's test are sensitive to the normality of the data and have not been considered in this study. The Brown–Forsythe test is more suitable for the test of heavy-tailed distribution than Levene's test because the former is not a statistical value using the usual average, but an end-of-tail average. Therefore, the statistical method chosen for our study was Levene's test (Conover et al., 2018). We use R programming to simulate the homogeneity. We first load a dataset from OSC webs (see section Data, part of data sources) and use the function “Levene test” to fit homogeneity. R programming will output homogeneity test results with *p*- values. Source codes for this are in Supplementary Materials at the end of the paper.

## Result

### Testing of the Hypothesis H1.1

Considering that some studies do not have special requirements for distribution, the initial 100 studies were screened before the normality test, and 87 studies that met the requirements were selected.

First, we sorted out two categories for these 87 studies: cognitive and social psychology according to Open Science Center (OSC) standard. There are 42 cases for cognitive psychology and 45 for social psychology, and the two areas have been roughly equal numbers. As for cross-area cases, we also follow the OSC division.

**Figure 1:**
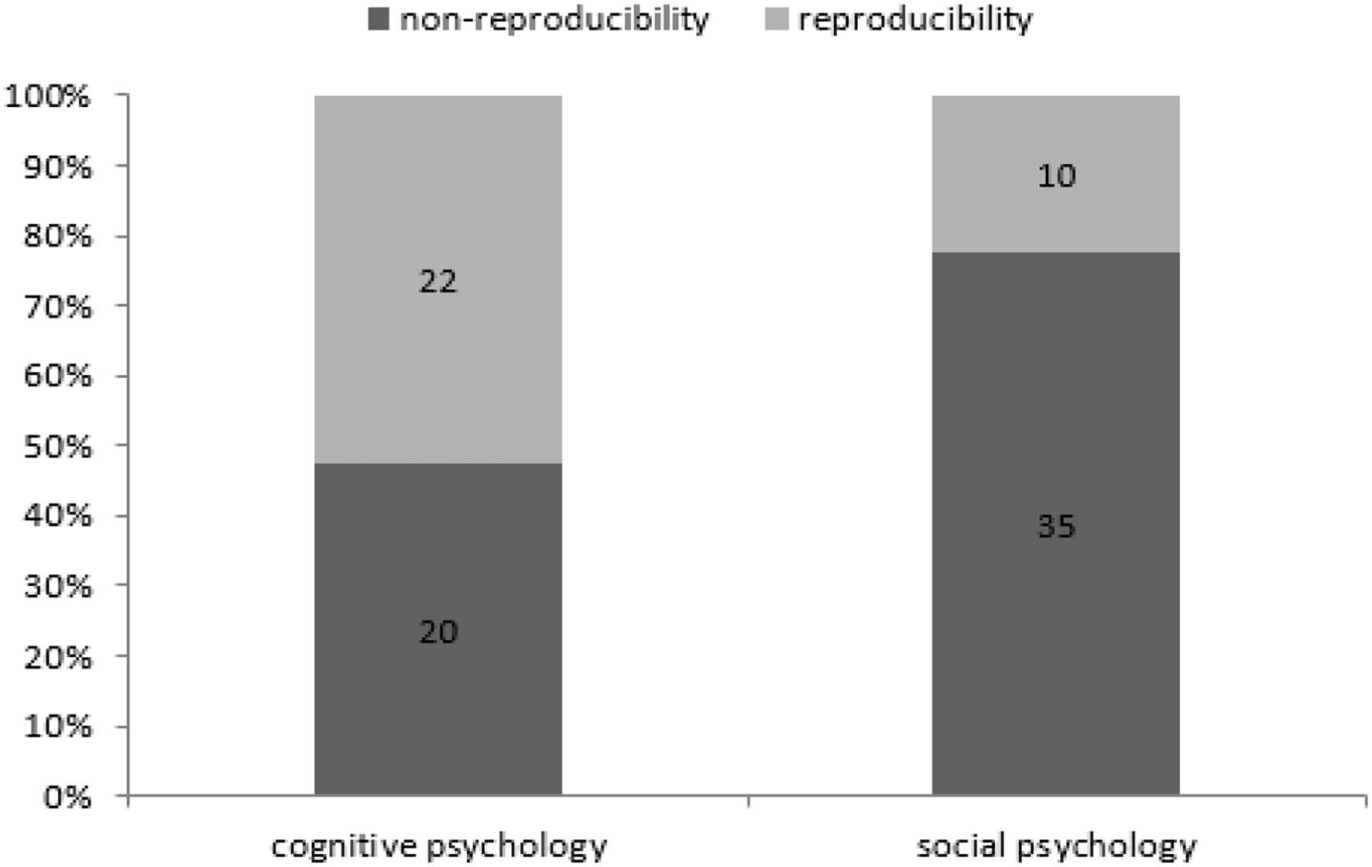
Reproducibility rate of cognitive/social psychology research.

Based on the above findings, the reproducibility of psychological research in different research areas was further calculated as shown in Figure 1. Cognitive psychology has 22 successful cases, 20 cases fail while the social psychology has 10 successful cases and 35 cases fail, and then the differences between these two research areas in reproducibility are very apparent. This figure also shows that the success probability of reproducibility in cognitive psychology is nearly 50% (47.6%), while the same rate is <25% (22.2%) in social psychology. Therefore, our hypothesis **H1.1** is verified, which says that the study of cognitive psychology is more likely to be reproduced than the study of social psychology. We also do fisher exact test for this hypothesis by taking the data as a 2 × 2 contingency table (*p* = 0.004231, alternative hypothesis: true odds ratio is not equal to 1, 95% confidence interval: 1.393561–10.925499, sample estimates: odds ratio = 3.786883). Taking significant level α = 0.05 > 0.004231, this make us accept alternative hypothesis. This claim is consistent with the one mentioned above using simple statistics.

### Testing of Normal Assumptions: Hypothesis H1.2 and H1.3

#### Method

**Figure 2:**
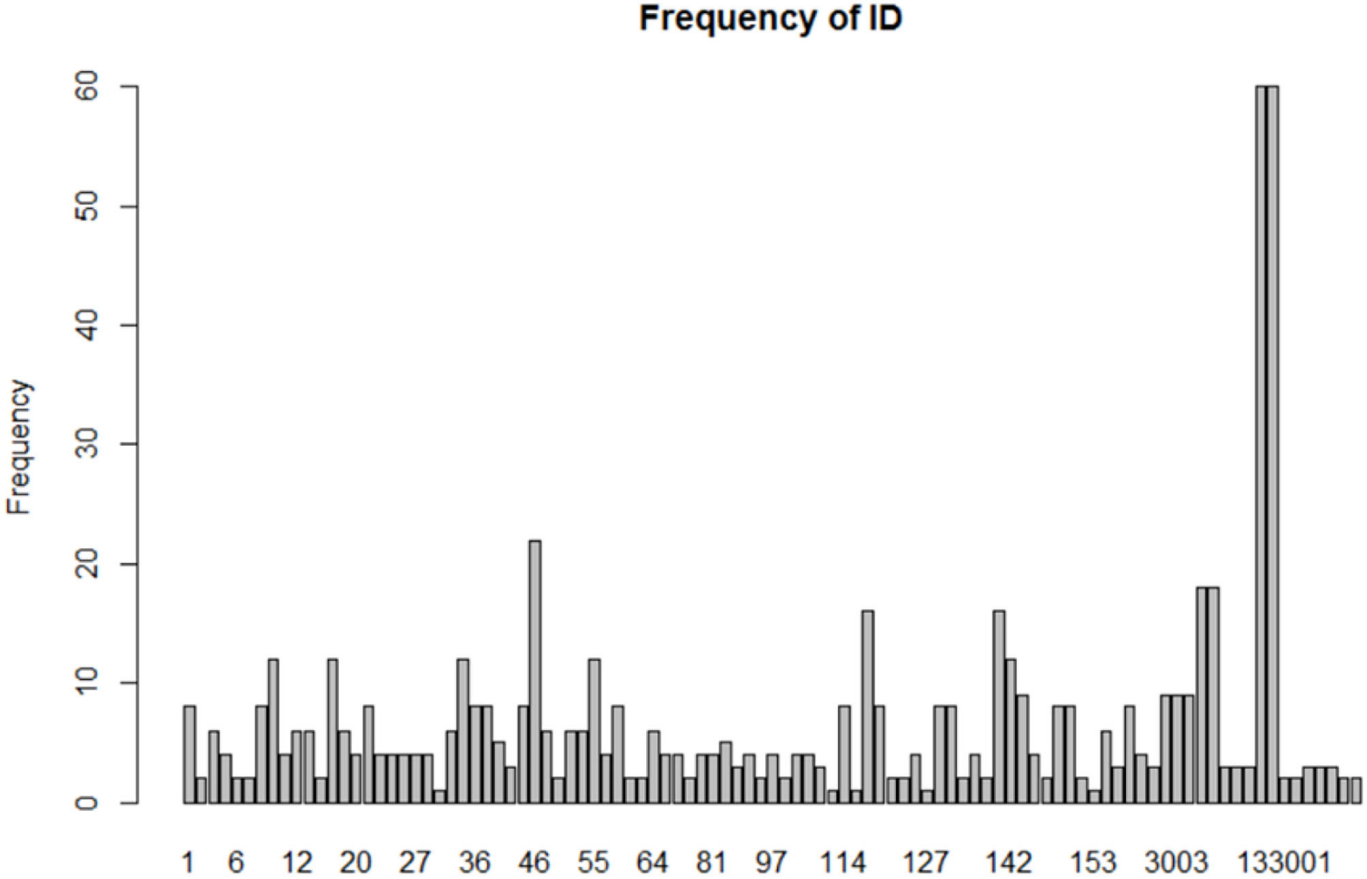
Frequency of the fitting of psychological variables in each study.

The reproducible research project team started with 167 psychology research papers and later selected 100 of these studies to implement replicated experiments. After sorting through these 100 studies, this paper eliminates several studies that use premises that do not require normality, such as chi-square analysis and qualitative variables; and then eliminate studies for which the original data could not be found or for which there was too little data to fit, leaving 87 studies. Each paper had an identifier number (ID), so the ID numbers ranged from 1 to 167. Each paper contained several psychologically significant variables, and for each variable, we fitted normality using the R statistical package, for a total of 650 variables. Each ID fit 6.57 times on average. The standard deviation was 8.72, and the median was 4. The smallest fit was 1 time, and the largest was fitted 60 times. Considering that for some studies, each variable in them has several different levels, and then we renumbered ID. The rule was that the original ID was appended to 001, 002, and 003. For example, if the original ID was 133, then the three psychological variables at different levels were numbered 133001, 133002, and 133003. It can be seen in Figure 2 that there are three very prominent 22, 60, and 60 fittings, corresponding to ID 46, 117001, and 117002, respectively. In the follow-up analysis, we have tried to treat the three different.

#### Normal Fittings in Different Study Areas of Psychology

Normality tests are used to determine the extent to which a data set conforms to a normal distribution, and how likely it is that the random variables under a given data set are normally distributed. There are roughly three ways to select specific tests, depending on the researcher's context (Razali and Wah, 2011):

(1) Descriptive statistics approach. The graphical or descriptive statistics are used to determine the extent to which a data set conforms to a normal distribution, the most common being the QQ plot.

(2) Null hypothesis testing based on frequency statistics. The difference between the actual and expected frequencies is used to test for normality, and then the null hypothesis is established to make a judgment. There may be differences in the data used in various tests, for example, the chi-square goodness-of-fit test groups the original data first and then compares the actual data with the theoretical frequencies, while Kolmogorov–Smirnov directly tests the original observed data, but there is no difference, in essence, both are based on frequency differences.

Graphical or descriptive statistics are used to determine the extent to which a data set conforms to a normal distribution, but there is no underlying variable to measure this extent, the most common being the QQ plot.

(3) Bayesian statistics. The Bayesian normality test approach does not use differences in data to measure normality, but rather uses the a priori parameters μ, σ to do so, comparing μ and σ of the actual data with μ and σ of the ideal distribution.

The normality hypothesis test uses the null hypothesis test. When *p* > 0.05, the distribution is considered to conform to the normal distribution, and when *p* < 0.05, the distribution does not conform to the normal distribution. Some rough statistics of 650 fittings of 87 studies are given in Table 2. This statistic is in favor of accepting hypothesis **H1.2** (not all psychological variables in the psychological studies meet the normality hypothesis). To strengthen this claim, we execute binom. test in R for the number of successes (we take cognitive psychology as an example: sample size = 110, number of trials = 282), the output is below: *p* = 0.5216, alternative hypothesis: true probability of success is >0.39, 95% confidence interval: 0.3415553–1.0000000, sample estimates: the probability of success = 0.3900709. But we can test H.1.2 in another way, pure logical reasoning. After fitting data in R, we can know by simple counting data how many percentages of data fitted are not subject to the normality (42.9% for cog. Psy, 48.7% for soc.psy, using α = 0.05).

**Table 2:** Normal fitting in different study fields of psychology.

**Study field**	**ID**	***P < * 0.05**	***P >* 0.05**	**Normality**
				**PCT**
Cognitive	—	161	121	42.9%
Social	—	116	110	48.7%
Social-special case	46	22	0	0%
Cognitive-special case	117001	16	44	73.3%
Cognitive-special case	117002	21	39	65%

**Figure 3:**
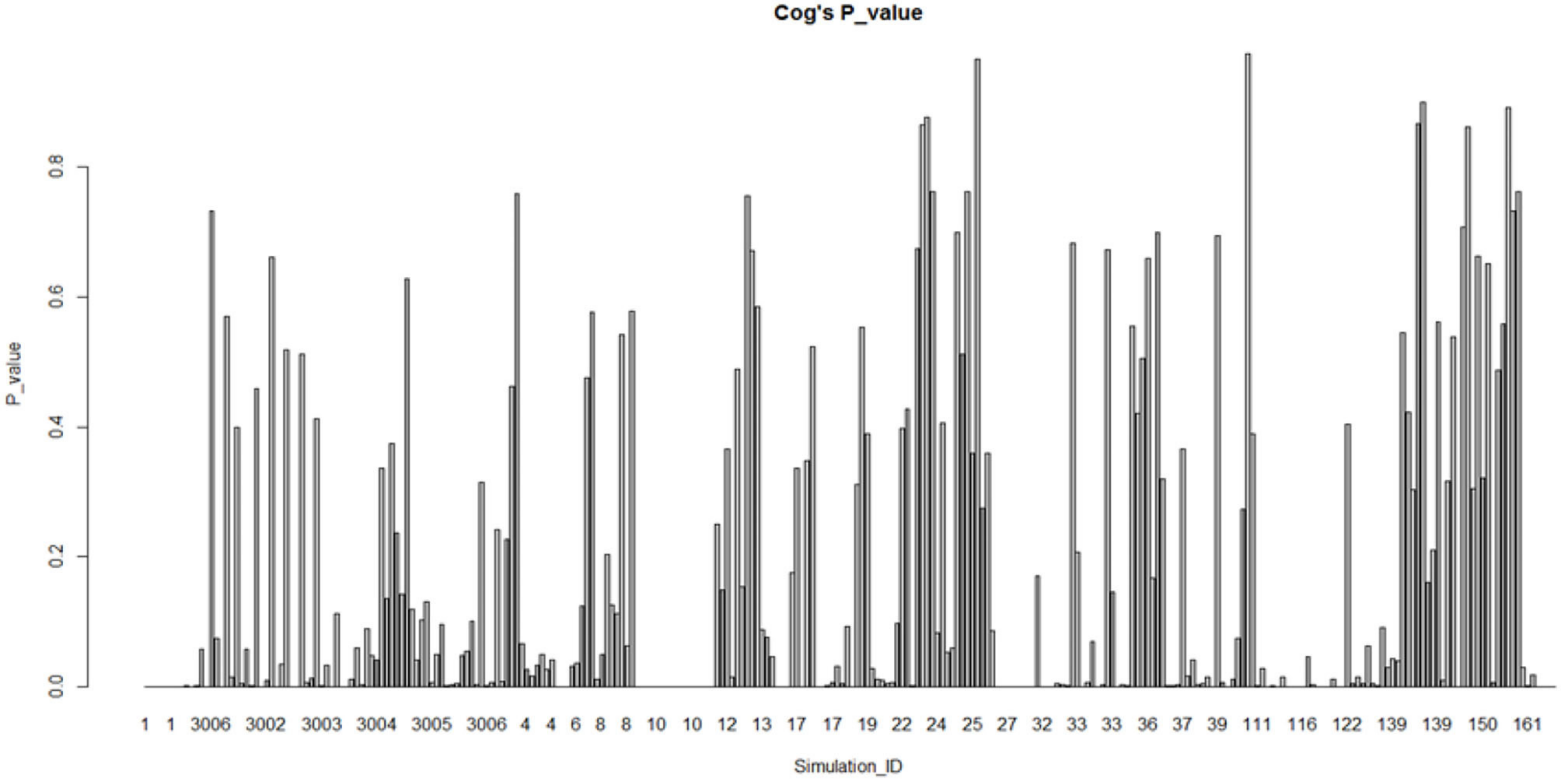
Fitting of cognitive psychology studies (remove fitting data ID 117001, 117002).

**Figure 4:**
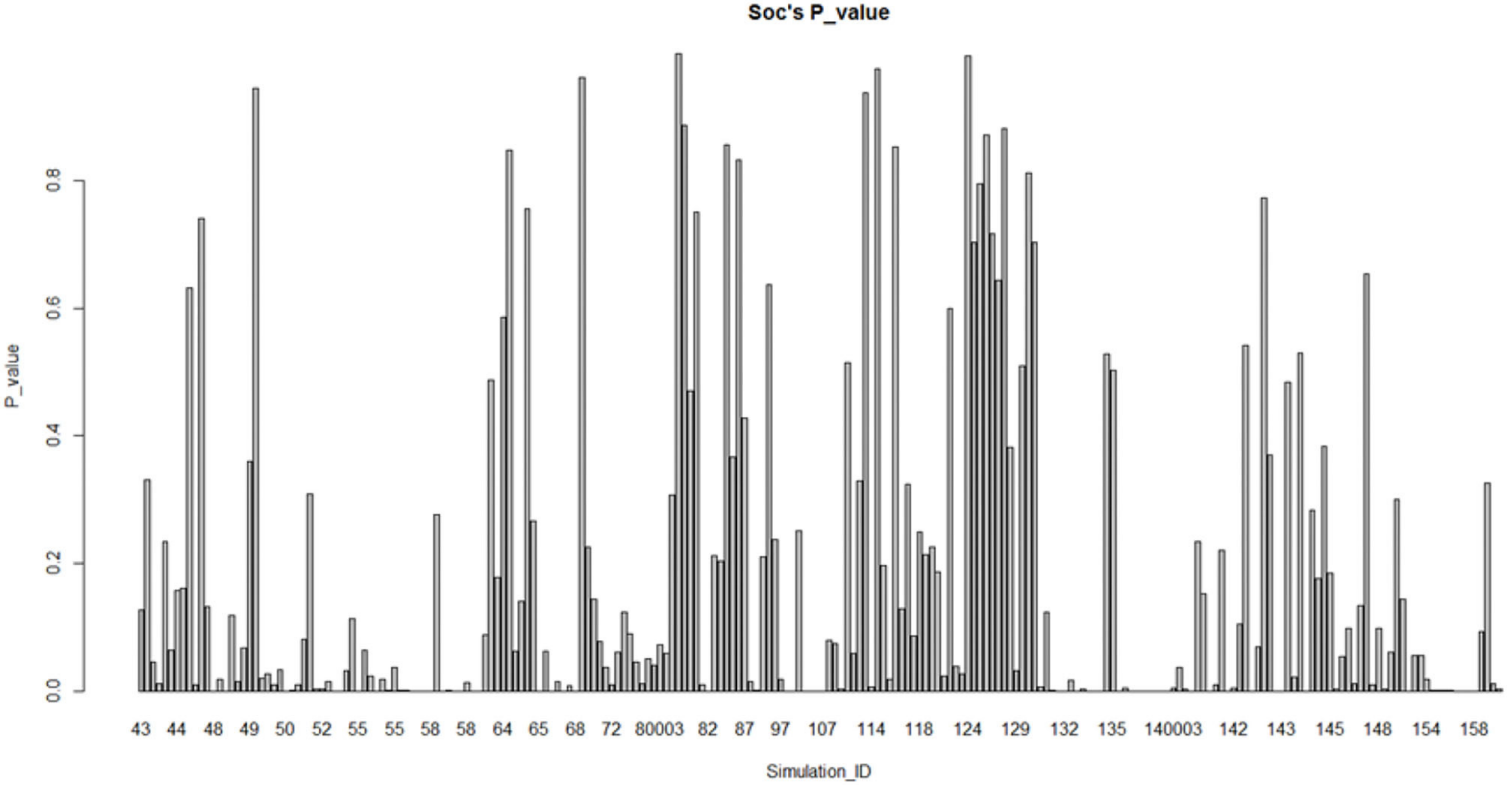
Fitting of social psychology studies (remove fitting data with ID 46).

The graphs based on the division of sub-fields in psychology are shown in Figures 3, 4. In the cognitive field (excluding the extreme ID 117001/117002), Most (57.1%) of them do not conform to the normal distribution. Relatively speaking, the research on social psychology (excluding the ID 46) does not conform to the normal distribution, which accounts for a smaller proportion (51.3%). Therefore, the normality comparison between these two sub-fields in psychology does not meet the assumption **H1.3**. We also do fisher exact test for this hypothesis by taking the data as a 2 × 2 contingency table (*p* = 0.2099, alternative hypothesis: true odds ratio is not equal to 1, 95% confidence interval: 0.8739768–1.8212425, sample estimates: odds ratio = 1.261163) Taking significant level α = 0.05 < 0.2099, this make us accept hypothesis: true odds ratio is equal to 1, which is consistent with the claim above.

However, from Table 2, we note that the normality test for fittings of the very special IDs, such as ID46, ID 117001, and 117002 were consistent with **H1.3** (the proportion of normal distribution in cognitive psychology was higher).

The use of *p*-value to test for normality seems inconsistent with the criticisms in this paper. This is correct. However, to prevent misuse of the *p*-value, here we use it as a heuristic strategy, which is judged in conjunction with other means such as intuition, which is exactly what the American Statistical Association (ASA) statement recommends. Apart from the *p*-value, the normality fit in this paper is judged in terms of how close it is to our intuition about the skewness of normality, that is, that the closer the shape parameter is to 0, the more normal (Jayalath et al., 2017).

### Testing of Hypothesis H2.1, H2.2, and H2.3

In this part, we examine the homogeneity of the variance hypothesis in the selected studies for which we selected ANOVA as the statistical method.

#### Robustness of t-test and ANOVA: Test for H2.1

The original data were 100 psychological studies. Considering that two studies used the same data source and for two studies we were unable to obtain the original data, our study counted the statistical methods and reproducibility rates of the remaining 96 studies, which are tabulated below (Table 3).

**Table 3:** Reproducibility in different statistical methods.

**Method**	**Reproduction**	**Reproduction**	**Reproducibility**
	**failure**	**success**	**rate**
*t*-Test	11	12	52.1%
ANOVA	41	20	32.8%
CHI	2	2	50%
Correlation	4	1	20%
Multilevel model	0	2	100%
HLM	0	1	100%

The table shows that the reproducibility of the studies using ANOVA at 32.8% is slightly lower than the average. While the studies used *t*-test have a higher reproducibility of 52.2%, far higher than the average level. Thus, hypothesis **H2.1** that the robustness of the *t*-test is greater than that of ANOVA is confirmed. We also do fisher exact test for this hypothesis by taking the data as a 2 × 2 contingency table ( = 0.1325, alternative hypothesis: true odds ratio is not equal to 1, 95% confidence interval: 0.1503008–1.3344524, sample estimates: odds ratio = 0.4516957). Taking significant level α = 0.05 < 0.1325, this makes us accept the hypothesis: true odds ratio is equal to 1. This contradicts the claim above for **H2.1** and needs further explanations.

#### Normality Hypothesis in ANOVA and Reproducibility: Test for H2.2

As we can see above, ANOVA and *t*-test are the two most used statistical methods in these studies. Since the *t*-test is more robust than ANOVA, we focus, in this section, on the impact of data assumptions in the *t*-test, which is the normality hypothesis, on reproducibility. The method of normal fitting used here is the same as in the previous section. A total of 61 studies were fitted 406 times since ID 117001, 117002, 46 fitting far more times (a total of 142). A separate list of inspections was made. Besides, in this case too, we have used *p* > 0.05 as the boundary value. The results are presented in Table 4.

**Table 4:** Normal distribution and reproducibility.

**Distribution**	**Unrepeatable**	**Repeatable**	**Repeatability rate**
Normal	138	53	27.7%
Non-normal	149	66	30.7%

We can see from this table that whether the normality hypothesis is satisfied or not is not very relevant to the reproducibility of the studies. So, hypothesis H2.2 cannot be verified. The fitted results of the three special cases excluded in the primary analysis showed that ID46 was not conforming to the normal distribution in all, ID 117001 and ID 117002 own 26.7 and 35.0% conformed to the normally distributed, but all the three studies were repeated successfully. Therefore, this did not support hypothesis **H2.2**. We also do fisher exact test for this hypothesis by taking the data as a 2 × 2 contingency table (*p* = 0.5851, alternative hypothesis: true odds ratio is not equal to 1, 95% confidence interval: 0.7344252–1.8152152, sample estimates: odds ratio = 1.152943). Taking significant level α = 0.05 < 0.5851, this makes us accept the hypothesis: true odds ratio is equal to 1. This claim is consistent with the one above.

#### Homogeneity of Variance Hypothesis in ANOVA and Reproducibility: Test for H2.3

**Figure 5:**
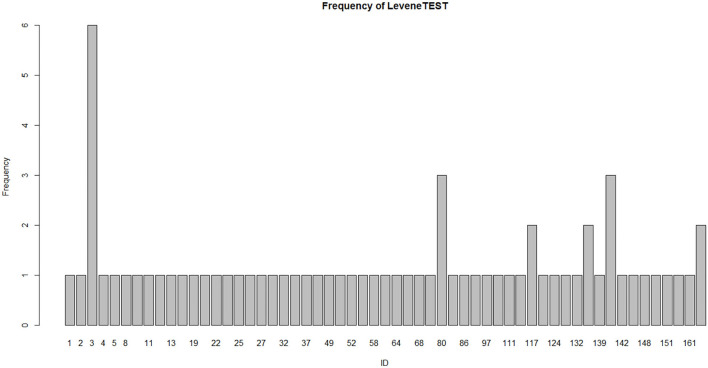
Fitting times of variance homogeneity test in 61 studies.

We conducted the homogeneity of variance test for 70 studies using Levene's test in 61 studies. The profile is shown in Figure 5. Source codes are appended to the end of the paper.

In this case, too, we still selected 0.05 as the boundary value. When *p* > 0.05, it accepted the null hypothesis, which indicates that the variance is homogeneous. When *p* < 0.05, it rejected the null hypothesis, which indicates that the variance is not homogeneous. The result of the variance homogeneity test is shown in Table 5.

**Table 5:** Variance homogeneity and reproducibility.

	**Repeatable**	**Unrepeatable**	**Repeatability rate**
Unequal variance	13	12	52%
Equal variance	7	38	15.6%

According to the result mentioned above, 64.3% of studies conformed to the variance homogeneity hypothesis and 35.7% did not conform to the hypothesis. For those who conformed to the variance hypothesis, the proportion of successful repeats was 15.6%, and for those who did not conform to the variance hypothesis, the proportion of successful repeats was 52.0%. This means that in terms of the satisfaction of the variance homogeneity hypothesis, not only did this finding fail to predict the reproducibility of the psychological research, but it also showed a reverse correlation, which is completely contrary to hypothesis **H2.3**. We also performed the Fisher exact test for this hypothesis by taking the data as a 2 × 2 contingency table (*p* = 0.002105, alternative hypothesis: true odds ratio is not equal to 1, 95% confidence interval: 1.674873–21.308843, sample estimates: odds ratio = 5.707206). Taking significant level α = 0.05 > 0.002105, this makes us accept the alternative hypothesis: the true odds ratio is not equal to 1. This claim is consistent with one using simple statistics.

## Discussion

In summary, our study aims to explore the factors that affect the reproducibility of psychological studies. Considering the important role that statistical methods play in psychological studies, we conducted an exploratory analysis with the help of data mining to test the effects of statistical presuppositions on the reproducibility of psychological studies.

We test the reproducibility of the studies belonging to different fields of psychology. The results of our analysis results show that the successful reproduction of studies in psychology is related to the fields that these studies belong. More specifically, studies in cognitive psychology were more likely to be repeated than those in social psychology. The normality test of the variables in psychology found the following: (1) The variables in psychological studies do not generally conform to the normal distribution, (2) To some extent, the larger the sample size, the greater is the probability of not conforming to the normal distribution. This indicates that we cannot generally think that all the variable data in psychological studies conform to normal distribution. Of course, whether the data conforms to the normal distribution or not, in addition to the properties of their own, also has a lot to do with the measurement method. This is so because the measurement method determines the form of the data to start with. For example, the data obtained from structured scales will be very different from the data obtained from the instruments of an experiment.

By analyzing the statistical methods used in psychological studies, we found that the studies using correlation analysis showed a low reproducibility rate. Considering that correlation analysis is more frequently used in social psychology, this finding reminds us of the following two points: (1) using correlation analysis as a preferred statistical method with cautions and (2) when citing a study using correlation analysis or applying its findings, it is necessary to pay greater attention to its scope of application and reliability. Because of only five data, one can be sure that this carefulness for correlation analysis has little to do with the robustness of the correlation analysis itself; however, it is worth investigating the further.

Further, because the hypotheses H2.3 and H2.4 are not statistically supported, it is not concluded that in terms of the robustness of ANOVA, the influence of normality is greater than the variance homogeneity. For the 61 studies covered in this regard, there is no correlation or effect between the normality hypothesis and the success of replication of these psychological studies, while the variance homogeneity is even negatively related to whether the repetition was successful. This finding implies that variance homogeneity has the resilience to reproducibility, which is related to its robustness (Trafimow, 2019b).

The study on reproducibility in psychology may conduct from an effective sample size and effective hypothesis tests based on the Bayesian manner. To enlarge samples, we can wait for elaborated and accumulated reproductivity data by the Open Science Collaboration, on other hand, we can adopt another strategy, for example, treating outcomes from unpublished studies as missing data, as suggested by Johnson et al. (2017). This work used the moment model and normal model to estimate *p*-values and missing tests or unpublished statistical tests based on the size of the original test 73. The results are that for the moment model, the missing test size (posterior mean) is 706 [95 %CI is (495, 956)], while for the normal model the size (posterior mean) is 669 [95% CI is (447, 925)]. This result indicates that about 90% of hypothesis tests are intentionally neglected for the publication preference. So, we think that maybe these work on sample sizes and the Bayesian way support this opinion that publication biases based on *p*-values caused the observed rates of non-reproducibility.

Our research has important implications as it indicates that the statistical assumptions for data certainly significantly influence the reproducibility of psychological studies and that one must unveil the assumptions behind the data before undertaking reproducible studies, and clarify complicated relations between statistical assumptions and reproducibility, not just pre-registering (Lurquin et al., 2016).

Our study also had several limitations. First, we did not consider the weight issues in the statistical simulations when analyzing the data fitting results. The weights of multiple fittings in the same study were not assigned in the process of normal fitting and there are some differences in the number of fittings in different studies. Some studies may have more than a dozen fittings, while some have only one fitting. This research did not have the different weight ratios for these studies at the time of the final integration, the number of participants in different studies was different, and the results of the fitting may have been affected by the number of participants. If a reasonable weight ingress is applied, then the data analysis results will support the original research assumptions in this study.

Second, our study needs a more systematic exploration of reproducibility. In our study, the reproducibility of psychological research is based on the data, but it ignores an examination of the source of the data. To some extent, the way data is extracted determines its form and property. For example, the Likert scale, to a large extent, constrains the form of data. As a result, it always fails to reflect the actual distribution of psychological traits. Therefore, we suggest that similar studies in the future would do well to incorporate the study methods into the argument of reproducibility in research and analyze the impact of research methods such as questionnaires and experiments.

Third, an increase in the data sources may lead to different conclusions. Our research covers only 100 studies. This number is not sufficient in view of the wide range of research areas that exist in psychology (Johnson et al., 2017). At present, there are possible data sources for being available, for example, some journals ask authors to provide materials to be open for being examined and pre-registered. But only the Open Science Center (OSC) systemically and comprehensively maintains the source (100 study cases) that may be the best quality in availability to reproductivity study in psychology. The current reproducibility studies almost take OSC sources as a sample, because of high-quality sources are sparse.

Finally, we should pay attention to the model in use and the hidden hypothesis. We have questioned the *p*-value validity in a hypothesis test, so in sequent passages, we use simple and rough statistics to evaluate our hypothesis, while other popular tests, for instance, the Fisher exact test have been exploited for comparisons. Most of the comparisons are consistent, but a few are against each other in boundary cases. Three are many reasons for the disagreements and, one of the reasons may be insufficiency in sample sizes of reproducibility experiments. For the CDC data, this multiple test model estimates the rate of false directional claims at roughly 32% among studies with *p* < 0.05, which would be considered unacceptably high in most multiple testing applications. By contrast, among studies with *p* < 0.005, a lower threshold is proposed, and the multiple test model estimate drops to 7% with upper confidence bound of 18% (Hung and Fithian, 2020). The estimation for the number of replication series as multiple tests is at least 197–375 using the model in (Hung and Fithian, 2020), This calculation is roughly in the same magnitude as the lower bound estimated by the Bayesian model (Etz and Vandekerckhove, 2016; van Aert and van Assen, 2016). These estimations are quite different from the CDC's metrics and imply that the present replications are not very perfect and complicated, but have considered credibility. So, our study based on the CDC dataset is seriously treated with some trust. Also, this indicates that the formalism model in use matters in discussing related replication problems. We must declare hidden assumptions in various models in replication study for comparisons, including Bayesian models and even natural language processing approaches, like the topic model (Fei, 2018).

## Conclusions

One of the principles of this work is that statistical assumptions are a prerequisite for reliable reproducibility studies and that reproducibility insights can be obtained using data mining; Six hypotheses were then examined, relating to the nature of the experimental data, the differences between the various branches of psychology, and the reliability of the research methods. The potential recommendation of this work is that, in addition to pre-registration, attention should be paid to the basic statistical hypothesis set of the model to improve reproducible studies; At the same time, the study suggests that the nature of psychological objects is related to the complexity of reproducible studies and violating the statistical assumptions of the hypothesis test in psychology is not necessarily to produce low reproductivity. This work is conceptually like another work that called for carefully checking the common statistical assumptions in psychological articles and further doing it with sincerity (Hoekstra et al., 2012). Data in psychology are not of the same nature as data in physics, being full of subjectivity and uncertainty, and inference is also uncertain; for the moment, there is not much research in this direction, but the Neutrosophic Statistics by Smarandache s work is noteworthy for dealing with indeterminate data (unclear, vague, partially unknown, contradictory, incomplete, etc.), and fuzzy logic-style inference (Smarandache, 2014, 2018).

## Data Availability Statement

The original contributions presented in the study are included in the article/supplementary material, further inquiries can be directed to the corresponding author/s.

## Author Contributions

DF and BT contributed to conception of the study, performed the software analysis, and validated the study. DF contributed to methodology of the study, supervised the study, were responsible for the project administration of the study, and received the funding to support this study. BT and SY performed the data collection and analysis and wrote the first draft of the manuscript. WZ, BT, and SY investigated for the study. DF and WZ reviewed and edited the manuscript. All authors contributed to manuscript revision, read, and approved the submitted version.

## Funding

This research was supported by the Artificial intelligence RongTong project from the Academy of Humanities and Social Sciences (No. 2020AI002), Wuhan University.

## Conflict of Interest

The authors declare that the research was conducted in the absence of any commercial or financial relationships that could be construed as a potential conflict of interest.

## Publisher's Note

All claims expressed in this article are solely those of the authors and do not necessarily represent those of their affiliated organizations, or those of the publisher, the editors and the reviewers. Any product that may be evaluated in this article, or claim that may be made by its manufacturer, is not guaranteed or endorsed by the publisher.
